# Does urbanization ameliorate the effect of endoparasite infection in kangaroo rats?

**DOI:** 10.1002/ece3.8062

**Published:** 2021-08-26

**Authors:** Gizelle Hurtado, Ghislaine Mayer, Karen E. Mabry

**Affiliations:** ^1^ Department of Biology New Mexico State University Las Cruces NM USA; ^2^ Norris Natural History Museum University of California Santa Cruz Santa Cruz CA USA; ^3^ Department of Biology Manhattan College Riverdale NY USA

**Keywords:** body condition, *Dipodomys*, parasite, urbanization

## Abstract

Urban development can fragment and degrade remnant habitat. Such habitat alterations can have profound impacts on wildlife, including effects on population density, parasite infection status, parasite prevalence, and body condition. We investigated the influence of urbanization on populations of Merriam's kangaroo rat (*Dipodomys merriami*) and their parasites. We predicted that urban development would lead to reduced abundance, increased parasite prevalence in urban populations, increased probability of parasite infection for individual animals, and decreased body condition of kangaroo rats in urban versus wildland areas. We live trapped kangaroo rats at 5 urban and 5 wildland sites in and around Las Cruces, NM, USA from 2013 to 2015, collected fecal samples from 209 kangaroo rats, and detected endoparasites using fecal flotation and molecular barcoding. Seven parasite species were detected, although only two parasitic worms, *Mastophorus dipodomis* and *Pterygodermatites dipodomis*, occurred frequently enough to allow for statistical analysis. We found no effects of urbanization on population density or probability of parasite infection. However, wildland animals infected with *P*. *dipodomis* had lower body condition scores than infected animals in urban areas or uninfected animals in either habitat. Our results suggest that urban environments may buffer Merriam's kangaroo rats from the detrimental impacts to body condition that *P*. *dipodomis* infections can cause.

## Graphical abstract

We investigated the influence of urbanization on populations of Merriam's kangaroo rat (*Dipodomys merriami*) and their parasites. We found no effects of urbanization on population density or probability of parasite infection. However, wildland animals infected with *Pterygodermatites dipodomis* had lower body condition scores than infected animals in urban areas or uninfected animals in either habitat, suggesting that urban environments may buffer Merriam's kangaroo rats from the detrimental impacts to body condition that *P. dipodomis* infections can cause.

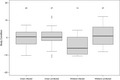

## 1 INTRODUCTION

Urbanization and associated urban, ex‐urban, and peri‐urban development are expected to increase worldwide to accommodate an increasing human population (McKinney, 2006). Urban development fragments landscapes and isolates remnant habitat patches (Bender et al., 1998; Debinski & Holt, 2000; Pickett & Thompson, 1978). By 2030, it is estimated that 1.2 million km^2^ will be under urban development worldwide, with the majority of new urban and ex‐urban development occurring in wildland areas (Seto et al., 2012). In the United States, the majority of urban growth is in wildlands in the southwestern and southeastern parts of the country, which are relatively undisturbed or undeveloped (Miller, 2012; Theobald, 2005; York et al., 2011).

Conversion of wildlands into urban environments can impact wildlife ecology. For example, wildlife abundance can be influenced through direct mortality (Fahrig & Rytwinski, 2009): small mammals and birds have increased mortality rates due to domestic cats (Loss et al., 2013), and roads account for a large portion of mortality for some mammal species (e.g., mountain lions; Schwab & Zandbergen, 2011; Vickers et al., 2015). Negative impacts of urbanization are sometimes sublethal and difficult to detect, particularly when wildlife populations persist in an area rather than experiencing large declines in population size or local extinction (Birnie‐Gauvin et al., 2016; Giraudeau et al., 2014; Valcarcel & Fernández‐Juricic, 2009; Zanette et al., 2011). In some cases, wildlife respond to disturbances from vehicles, humans, and domestic animals as a perceived risk or as a perceived competitor, spending time and energy responding to these disturbances instead of foraging (Shier et al., 2012; Valcarcel & Fernández‐Juricic, 2009; Zanette et al., 2011). This decrease in foraging and increase in energy expenditure can lead to reduced food provisioning for young and reduced reproduction (Bonnington et al., 2013; Zanette et al., 2011). For example, an experimental study showed that blackbirds (*Turdus merula*) exposed to a domestic cat model exhibited decreased care for young (Bonnington et al., 2013).

However, the effects of urbanization on wildlife are not necessarily negative. Urban areas also offer potential benefits such as readily available urban food sources (e.g., garbage, compost piles, ornamental/fruit trees, bird feeders, and pet food), and urban denning and roosting opportunities (e.g. urban planted trees, gardens, and basements; Becker et al., 2015; Oro et al., 2013). These anthropogenic resources provide opportunities for wildlife, and may increase urban wildlife populations as compared to wildland habitats. For example, raccoons and foxes can have higher population densities in urban areas, and foxes had decreased mortality in urban versus wildlands; this has been associated with anthropogenic food sources (Oro et al., 2013; Prange et al., 2003; Recio et al., 2015; Riley et al., 1998).

Both urbanization and parasite infection may affect body condition of wild animals, and responses to urbanization can be complex (Murray et al., 2019). Evidence from a variety of species indicates that urbanization can lead to decreased body condition (Hellgren & Polnaszek, 2011; Lomas et al., 2015; Murray et al., 2019; Ware et al., 2015). In addition, anthropogenic food sources are sometimes of relatively low nutritional quality, which may place wildlife in a nutrient‐deficient state and influence maintenance and reproductive capability (Birnie‐Gauvin et al., 2016; Oro et al., 2013; Plummer et al., 2013). Further, wildlife that are infected with parasites can have decreased body condition as compared to non‐infected animals (Debeffe et al., 2016; Stien et al., 2002; Vandegrift et al., 2008). Some parasite‐infected wildlife experience decreased reproduction (Altizer et al., 2003; Gooderham & Schulte‐Hostedde, 2011; Hudson, 1986; Vandegrift & Hudson, 2009; Watson, 2013), which can lead to population declines. Importantly, urbanization and parasite infection may have interactive effects on wildlife (Murray et al., 2019): a variety of parasites and disease‐causing agents have been detected in animals living in urban and suburban environments, including viruses, bacteria, and endoparasites (Adam et al., 2016; Clinton et al., 2010; Gordon et al., 2016; Korpe et al., 2016; Sibley et al., 2009). Some of these disease‐causing organisms are transmittable to humans (i.e., zoonotic) and/or livestock and domestic animals.

Due to the increased growth of urban development, it is important to understand how wildlife are impacted by expanding urban areas. These expanding urban areas can impact wildlife abundance, parasite prevalence, and body condition; urban environments may also facilitate the interaction of parasite infection and other potential stressors, exacerbating their impacts on wildlife. We investigated the effects of urbanization on population density, parasite presence and prevalence, and body condition in Merriam's kangaroo rats (*Dipodomys merriami*). Kangaroo rats, which are granivorous and largely solitary, are a highly suitable study group in which to examine the influence of urban development on wildlife ecology and disease. These rodents are widespread throughout the western United States and are found in both wildland and urban environments (DaVanon et al., 2016; Germaine et al., 2001). A variety of both endoparasites and ectoparasites have been documented in Merriam's kangaroo rat (Decker et al., 2001; Ford et al., 2004; Holdenried & Quan, 1956; Iturbe‐Morgado et al., 2017; King & Babero, 1974; Martínez‐Salazar et al., 2016; Stout & Duszynski, 1983), and kangaroo rats may be involved in the enzootic maintenance of zoonotic parasites and diseases (Antolin et al., 2002; Decker et al., 2001; Ford et al., 2004; Holdenried & Quan, 1956; King & Babero, 1974). *D*. *merriami* has also been identified as a potential hyper‐reservoir (a species that carries two or more zoonoses) for zoonotic diseases (Han et al., 2015). We predicted that kangaroo rats would have a lower population density, increased parasite infection, increased parasite prevalence, and decreased body condition in urban versus wildland habitats.

## 2 MATERIALS AND METHODS

### 2.1 Study area

**FIGURE 1:**
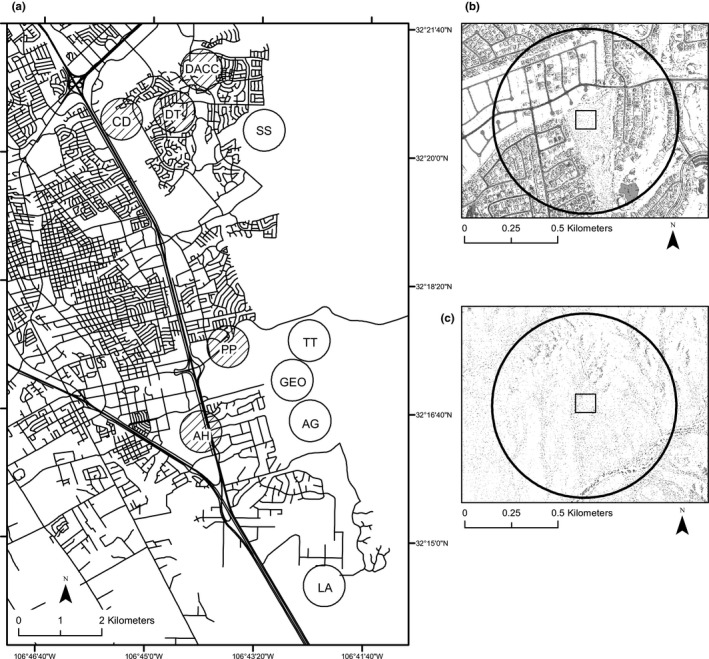
(a) Map of Las Cruces, NM and surrounding wildlands (modified from Hurtado & Mabry, 2017). Circles are the 500 m buffers around trapping sites that were used to create the urbanization index, squares the 100 m by 100 m trapping sites. Cross‐hatched buffers are urban areas and non‐shaded buffers are wildland areas. Examples of urban (b) and wildland (c) trapping sites

We conducted this study in and around the City of Las Cruces, New Mexico, USA (32°19′35.7414″, −106°46′31.569″; Figure 1; modified from Hurtado and Mabry (2017)). Las Cruces is a growing urban area: the total human population of Las Cruces increased by >25% from 2000 to 2014 (74,267 to 101,408; U.S. Census Bureau, 2015). Las Cruces encompasses several urban parks and open spaces with natural vegetation and is surrounded by undeveloped desert (Bureau of Land Management lands). The study area is part of the Chihuahuan desert ecoregion and the climate is characterized as arid or semi‐arid, with peak rainfall occurring during summer monsoons with smaller secondary rain events during the winter months. Mean annual temperature is 14.70°C and mean annual precipitation is 245.1 mm (Havstad et al., 2006). Typical vegetation includes mesquite (*Prosopis glandulosa*), creosote (*Larrea tridentata*), ocotillo (*Fouquieria splendens*), yucca (*Yucca baccata, Y. elata, Y. treculeana*), purple pricklypear (*Opuntia macrocentra),* scarlet hedgehog cactus *(Echinocereus coccineus)*, portulaca (*Portulaca* spp), limoncillo (*Pectis papposa*), zinnia (*Zinnia acerosa*), roundleaf buckwheat (*Eriogonum rotundifolium*), and various grasses, including *Panicum* spp*, Bouteloua barbata, B. aristidoides,* and *Dasyochloa pulchella*.

### 2.2 Site characteristics

We established sites that were similar in native vegetation in urban (*n* = 5 within Las Cruces city limits) and wildland environments (*n* = 5 on federal and state properties surrounding Las Cruces; Table 1), and that were large enough to accommodate a 1 ha trapping grid. Wildland sites were located ≥500 m from paved roads, and all study sites were at least 1 km from each other (Figure 1). Eight of the sites were characterized in Hurtado and Mabry (2017), with two additional sites added here (DACC and SS). Methods for characterizing vegetation at study sites follow Hurtado and Mabry (2017). Line‐point intercept transects were used to quantify vegetation cover at all sites. Six 50‐m line‐point intercepts were randomly dispersed across each site (total of 300 m surveyed per site). To compare the percent cover by grasses, shrubs, forbs, bare ground, and rocks between urban and wildland sites, we used Wilcoxon rank sums tests. An urban index for the study area was created using Landsat Thematic Mapper (LTM) imagery at the 30‐m spatial resolution (http://www.ngdc.noaa.gov/metadata). To quantify the degree of urban development surrounding each site, we measured the proportion of pixels within 500 m of the center of each study site that represented impervious surface, which tends to be materials associated with urbanization, such as roads, cement, and buildings, and serves as a proxy for urbanization (Figure 1; see also Hurtado and Mabry (2017) for details). To verify the urbanization index, we counted all the buildings at a site (housing units and commercial units) within a 1‐km buffer. All processing was conducted in ArcGIS 10.1 (ESRI). There was a strong correlation between the number of buildings at a site and the urban index (Pearson's correlation: *r* ^2^ = .93, *t*_8_ = 7.15, *p* < .01), indicating that the urban index we created was a good measure of urbanization. A Wilcoxon rank sums test was used to compare the urban index between urban and wildland sites.

**TABLE 1:** Descriptions of 5 urban and 5 wildland study sites in and around Las Cruces, NM, USA (modified from Hurtado & Mabry, 2017)

Site name	Latitude	Longitude	Site type	Urban index
Arrow Head (AH)	32.273	−106.736	Urban	0.31
Copperstone Dam (CD)	32.341	−106.758	Urban	0.30
DACC East Mesa (DACC)	32.351	−106.738	Urban	0.33
Desert Trails (DT)	32.343	−106.744	Urban	0.38
Park Place (PP)	32.292	−106.730	Urban	0.35
Aggie Rodeo (AG)	32.276	−106.709	Wildland	0.05
Geothermal (GEO)	32.283	−106.713	Wildland	0.04
Las Alturas (LA)	32.243	−106.704	Wildland	0.02
Sonora Springs (SS)	32.341	−106.721	Wildland	0.02
Two Towers (TT)	32.295	−106.709	Wildland	0.02

### 2.3 Live trapping

Merriam's kangaroo rats were live trapped from May to November 2013, June to September 2014, and May to October 2015. Data for abundance estimates were collected during 2013; data on parasite infection were collected during all 3 years. In 2013, 10 trapping grids, each 100 m by 100 m with 10 m spacing between traps, were established: 5 in urban and 5 in wildland habitats. In 2013, 100 traps were set on each grid for 3 consecutive nights for a total of 3,000 trap nights. In 2014 and 2015, traps were instead placed along trap lines in locations likely to maximize captures (e.g., near burrows and kangaroo rat trails) located within the perimeter of established grids. Sherman live traps (model XLKGDT; H.B. Sherman Co., Tallahassee, FL) were baited with a mix of millet and sunflower seed, set shortly before sunset, and checked at or before sunrise the next morning. Each captured Merriam's kangaroo rat was uniquely marked with numbered Monel ear tags (National Band and Tag, Newport, KY) and standard data were recorded and samples collected (e.g., sex, reproductive condition, foot length, mass, and fecal samples) before release at the site of capture. All research procedures were consistent with the guidelines of the American Society of Mammalogists (Sikes et al., 2011) and conducted under an approved New Mexico State University IACUC protocol (protocol 2013‐014).

### 2.4 Abundance and density estimates

We estimated population size of kangaroo rats in urban and wildland areas in 2013 using closed population Huggins p and c models in Program MARK (White & Burnham, 1999), using Akaike's Information Criterion corrected for small sample size (AICc) to determine the most parsimonious model of the influence of urbanization on kangaroo rat abundance. We used the Huggins model because we assumed that the kangaroo rat populations at our study sites approximated closed populations over our relatively short trap periods (3 nights). We tested for differences in capture (p) and recapture (c) probabilities between urban and wildland sites. Two sites, 1 each from urban (AH) and wildland (TT), were dropped from the analysis due to low captures (only 3 individuals were captured at either site in 2013). The model averaging function in Program MARK was used to estimate population size, and population density was calculated by dividing the estimated abundance in urban or wildland by 4 ha, the total area trapped in each habitat type.

### 2.5 Parasite presence

Not all animals captured had associated fecal samples; only animals that had associated fecal samples were tested for parasite infection and used in further analysis. Endoparasites present in fecal samples were identified by fecal flotation and molecular barcoding. Fecal samples were collected in the field and placed in 2‐ml microfuge tubes labeled with each animal's unique tag number. All fecal samples were stored at −20°C until analysis. Samples were shipped on ice to both the Colorado State University Veterinary Diagnostic Laboratory (CSU‐VDL) and the Mayer lab for identification. Samples taken from kangaroo rats in 2013, 2014 and 2015 were sent to CSU‐VDL for detection of parasites using a modified fecal flotation technique with double centrifugation and a sugar solution (with a specific gravity of 1.27). Endoparasite presence was determined by the detection of eggs, cysts, and oocysts. Parasites were determined by comparison with parasites known to be carried by kangaroo rats or from original descriptions of parasites. In some cases, parasites were not able to be sporulated and could not be identified beyond genus (Lora R. Ballweber, personal communication).

A subset of samples from 2013 and 2015 were evaluated for endoparasites via molecular barcoding; *G. lamblia* and *C*. *parvum* were chosen because of their potential to cause disease in humans. DNA was extracted from kangaroo rat fecal samples and purified using the Qiagen Stool DNA kit (Qiagen, Valencia, CA). The DNA extraction was done according to the manufacturer's protocol (Qiagen, Valencia, CA). Nested PCR was performed to detect *G. lamblia* using primers that target the β‐giardin gene (Cacciò et al., 2002). The forward primer for the first reaction was Gia7 (5′‐AAGCCCGACGACCTCACCCGCAGTGC‐3′) and the reverse primer was Gia759 (5′‐GAGGCCGCCCTGGATCTTCGAGACGAC‐3′). In the first step, the conditions were 94°C for 5 min, 94°C for 30 s, 55°C for 30 s, and 72°C for 1 min. For the second step, a 511 bp fragment was amplified using the Gia 7 nested forward primer and the Gia 759 nested reverse primer was used. Conditions were set at 95°C for 5 min, 95°C for 30 s, 65°C for 30 s, 72°C for 1 min, and 72°C for 7 min. The second set of primers was the following: Gia7 nested forward (5′‐GAACGAACGAGATCGAGGTCCG‐3′) and reverse Gia 759 nested reverse (5′‐ CTCGACGAGCTTCGTGTT‐3′). *C*. *parvum* DNA was detected by using the LAX primer pairs LAX469F (5′‐CCGAGTTTGATCCAAAAAGTTACGAA‐3′), and LAX869R (5′‐TAGCTCCTCATATGCCTTATTGAGTA‐3′; Laxer et al., 1991; Rochelle et al., 1997). Cycling conditions were 94°C for 3 min, 94°C for 45 s, 52°C for 45 s, 72°C for 1 min, and 72°C for 7 min. All PCR reactions were performed in a final volume of 25 µl, which included 1 µl of the extracted genomic DNA. The PCR products were purified and sequenced by Sanger sequencing by Genewiz (South Plainfield, NJ). Sequences were assembled using Sequencher 5.3. The nucleotide sequences were aligned with reference sequences from GenBank and analyzed using BLAST (https://blast.ncbi.nlm.nih.gov/Blast.cgi ).

### 2.6 Parasite prevalence

Parasite prevalence (number infected/number tested) was determined for all 10 sites (Jovani & Tella, 2006). The number of individuals tested from each site ranged from 8 to 42, with mean ±1 *SE* = 20.10 ± 3.08. A sample size of 10–20 decreases uncertainty in estimates of prevalence, without losing data to low sample cut‐offs (Jovani & Tella, 2006). Prevalence of infection by habitat (urban vs. wildland) was compared using a Wilcoxon signed rank test.

### 2.7 Relationship between individual infection status and habitat

We determined if habitat affected individual infection status using binomial generalized linear mixed models (GLMMs) implemented in the R package lmerTest (Kuznetsova et al., 2017). Infection was scored as presence/absence of each parasite species for each individual kangaroo rat. We ran separate binomial GLMMs for infection with *Pterygodermatites dipodomis* and *Mastophorus dipodomis*, with habitat as a fixed factor and site and year as random factors.

### 2.8 Body condition

Body condition was assessed by taking the residuals of the regression of body mass on foot length (only adult males ≥30 g in body mass were used in this analysis to avoid the potentially confounding effects of undetected pregnancy on estimates of female body condition; Schulte‐Hostedde et al., 2005). Males were identified via external sexual characteristics. The fixed effects of habitat (urban vs. wildland) and parasite infection (infected vs. uninfected) and the interaction between habitat and parasite infection on body condition were assessed using a GLMM with random effects of site and year implemented using the R package lmerTest (Kuznetsova et al., 2017). All statistical tests were conducted in R 3.1.2 (R Development Core Team, 2015), with *α* = 0.05.

## 3 RESULTS

### 3.1 Site characteristics

There was no difference between urban and wildland sites in any measured environmental variables other than the urban index (Table 2); there was a higher proportion of impervious surface in urban sites than in wildland sites.

**TABLE 2:** Mean (±1 *SE*) urban index and percent cover for urban and wildland sites (*n* = 5 of each habitat type) in and near Las Cruces, New Mexico, USA, with the Wilcoxon test statistic (*W*) and *p*‐value for between‐habitat comparisons (modified from Hurtado & Mabry, 2017)

Variable	Urban		Wildland		*W*	*p*
	Mean	*SE*	Mean	*SE*		
Urban index	0.33	0.01	0.03	0.01	25.00	.**01**
% Shrub	5.50	0.45	5.56	0.40	12.00	1.00
% Forb	0.39	0.19	0.81	0.24	6.00	.22
% Grass	0.24	0.17	0.78	0.45	9.50	.58
% Bare ground	57.67	6.24	46.87	6.28	18.00	.30
% Litter	10.46	3.82	8.87	1.13	12.5	1.00
% Rock	8.60	4.94	11.4	5.81	9.00	.54

Note

Statistically‐significant *p*‐value is bolded.

### 3.2 Abundance and population density

We found equal support for the null model of no effect of habitat on either capture (p) or recapture (c) probability and a model that included different values for p and c within habitats (Table 3). An additional model, which included habitat‐specific values for both p and c (p[habitat] ≠c[habitat]), was dropped from consideration due to unreasonably large confidence intervals. We used weighted model averaging to estimate mean population size (±1 *SE*): urban =55.65 ± 5.53, 95% CI = 44.81–66.50, wildland = 57.98 ± 5.72, 95% CI = 46.76–69.19. Population density was approximately 14 individuals/ha in both habitat types.

**TABLE 3:** Results of Huggins p and c models in Program MARK used to determine the influence of urbanization on kangaroo rat abundance

Model	Model‐likelihood	Number parameters (*k*)	AICc	Delta AICc	Akaike weight	Deviance
{p(.) = c(.)}	1.000	1	375.622	0.000	0.458	590.800
{p(.) ≠ c(.)}	0.822	2	376.015	0.400	0.400	589.150
{p(habitat) = c(habitat)}	0.364	2	377.643	2.021	0.166	590.778

Note

Probability of capture (p), and recapture probability (c) constant (.), and not equal (/).

### 3.3 Parasite presence

Seven endoparasite species were detected in kangaroo rats via fecal flotation and molecular barcoding (Table 4), but only *Pterygodermatites dipodomis* (70 infected/201 tested) and *Mastophorus dipodomis* (41 infected/201 tested) were detected frequently enough to allow for statistical analysis. We detected 10 or fewer occurrences of all other endoparasite species (Table 4).

**TABLE 4:** Descriptive statistics for endoparasites detected

Parasite	Habitat	Number tested	Number infected	Prevalence
*Pterygodermatites dipodomis*	Urban	109	45	0.41
	Wild	92	25	0.27
*Mastophorus dipodomis*	Urban	109	16	0.15
	Wild	92	25	0.27
*Giardia lamblia*	Urban	114	7	0.06
	Wild	95	3	0.03
*Cryptosporidium parvum*	Urban	71	3	0.04
	Wild	54	1	0.02
*Eimeria* sp.	Urban	109	0	0.00
	Wild	92	3	0.03
*Heteromoxyuris* sp.	Urban	109	1	0.01
	Wild	92	1	0.01
*Catenotaenia* sp.	Urban	109	1	0.01
	Wild	92	1	0.01

Note

Parasite, parasite detected; habitat, habitat type in which kangaroo rats were captured, number tested, number of kangaroo rats tested for the presence of that endoparasite, number infected, the number of kangaroo rats that were infected with that parasite, prevalence, prevalence of each parasite in the urban or wild populations of kangaroo rats. In some cases, only genus is cited, and this is due to the dearth of information on the parasites detected.

### 3.4 Parasite prevalence

Parasite prevalence was not different between urban and wildland kangaroo rat populations for either *P*. *dipodomis* (Wilcoxon rank sums test, *T*_8_ = 17.5, *p* = .34, CI = −0.352–0.447) or *M*. *dipodomis* (Wilcoxon rank sums test, *T*_8_ = 7.00, *p* = .30, CI = −0.269–0.115; Table 4).

### 3.5 Relationship between individual infection status and habitat

We did not detect effects of habitat on an individual animal's probability of infection with either parasite. Overall, Merriam's kangaroo rats living in wildland habitats appeared to have a slightly lower rate of infection with *P*. *dipodomis* as compared to urban animals (Table 4), but the effect was not statistically significant (binomial GLMM: *N* = 201, *β* = −0.65 ± 0.84, *z* = −0.77, *p* = .44). Animals living in wildland habitats appeared to have a somewhat higher rate of infection with *M*. *dipodomis* as compared to those in urban habitats (Table 4), but again, the effect of habitat was not statistically significant (binomial GLMM: *N* = 201, *β* = 0.72 ± 0.47, *z* = 1.54, *p* = .12).

### 3.6 Body condition

**FIGURE 2:**
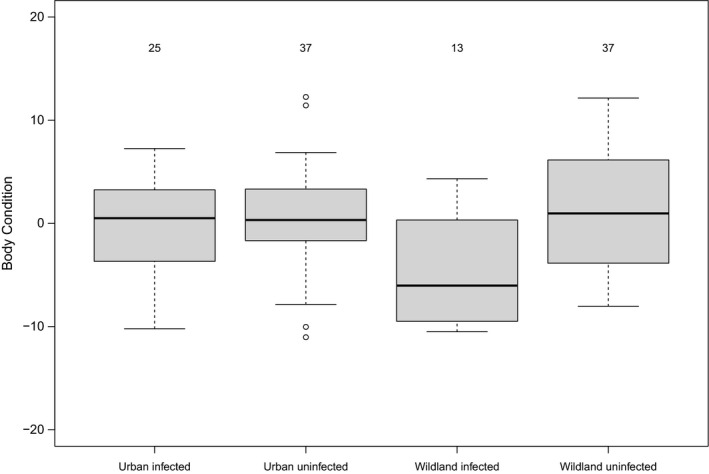
Body condition score and *Pterygodermatites dipodomis* infection in kangaroo rats in urban and wildland habitats, mean ± 1 *SE*. The box indicates first and third quartiles, and numbers over each box indicate sample size in each group. Thick lines indicate means. Body condition scores >0 indicate that animals are in better‐than‐average body condition and negative values mean that animals are in worse‐than‐average body condition

Contrary to our expectations, animals infected with *P*. *dipodomis* and living in wildland habitats had lower body condition compared to other kangaroo rats (GLMM: *N* = 112, habitat, *β* = 0.54 ± 1.40, *t* = 0.38, *p* = .71; infection, *β* = −0.87 ± 1.37, *t* = −0.64, *p* = .53; habitat × infection, *β* = −4.58 ± 2.16, *t* = −2.13, *p* = .04; Figure 2). Neither *M*. *dipodomis* infection nor habitat type affected kangaroo rat body condition (GLMM: *N* = 112, habitat, *β* = −0.76 ± 1.53, *t *= −0.50, *p* =.63; infection, *β* = 0.24 ± 2.05, *t* = 0.12, *p* = .91; habitat × infection, *β* = 0.88 ± 2.60, *t* = 0.34, *p* = .73).

## 4 DISCUSSION

We expected to find that urbanization would negatively affect Merriam's kangaroo rats, and predicted that populations in urban parks would have lower population density, higher parasite prevalence, a higher probability of parasite infection for individuals, and lower body condition than in populations in undeveloped desert habitats. Instead, we found no effect of urbanization on any variable examined, other than an interaction between urbanization and infection with the parasite *P*. *dipodomis* on body condition. Intriguingly, the direction of this effect was that infected animals living in urban parks had similar body condition scores to uninfected animals in both habitats, suggesting that conditions within urban habitats may ameliorate the expected negative effects of infection. Wildland animals infected with *P*. *dipodomis* exhibited decreased body condition, as expected from results of previous studies on white‐footed mice *Peromyscus leucopus* infected with a related parasite species (*P*. *peromysci*; Vandegrift & Hudson, 2009; Vandegrift et al., 2008). The negative effects of infection may be due to chronic immune stress, which may reduce body condition and decrease reproduction (Brooks & Mateo, 2013; Vandegrift et al., 2008). Further, *P*. *peromysci* infection can alter behavior and increase the likelihood of depredation for *Peromyscus* (Luong et al., 2011); however, we did not investigate the effects of infection on behavior in *D*. *merriami* due to sample size limitations. We observed no effect of *M*. *dipodomis* infection or habitat on kangaroo rat body condition. Other researchers have found differences in *Mastophorus* infection by habitat type, but similar to our results, they also found no difference in body condition by habitat (Lafferty et al., 2010).

Urban areas tend to maintain higher levels of plant productivity than surrounding wildland areas (e.g., active watering and nutrient inputs), and urban areas may moderate environmental fluctuations (e.g., heat islands, water runoff) as compared to surrounding wildlands (Faeth et al., 2005; Zhao et al., 2016). An increase in vegetation in urban areas may help kangaroo rats infected with *P*. *dipodomis* cope with parasite infection. Urbanization can influence processes at multiple ecological levels; for example, increased plant primary production could translate into effects on herbivores, predators, and parasites at higher trophic levels. We found no differences in percent cover by different functional groups of vegetation (grass, forbs, and shrubs) between urban and wildland sites, similar to the results of another study conducted in similar habitats in the same region (DaVanon et al., 2016). However, DaVanon et al. (2016) did find lower plant recruitment, higher herbivory rates, and higher mammal activity in urban areas, which may indicate that urban animals utilize resources differently than wildland animals. Specifically, increased foraging on anthropogenic resources in urban habitats may allow infected animals to maintain similar body condition as uninfected animals, leading to differential responses to parasitism in urban and wildland populations. Of potential relevance to human health, we detected *Giardia lamblia* and *Cryptosporidium parvum*, both of which can cause disease in humans, in 3%–4% of samples from urban Merriam's kangaroo rats.

We found no effect of urbanization on population density of Merriam's kangaroo rat. Further, our estimate of population density (~14 individuals/ha) was almost twice that reported by Lightfoot et al. (2012; 7.35/ha). It is possible that these differences are due to differences in sampling. Lightfoot et al. (2012) included 11 years of population data, whereas we have only one year (2013), which had higher than average population density. However, estimated population density in this study was within the range of densities recorded over 11 years (Lightfoot et al., 2012). One reason that we may not have found an effect of urbanization on population density is that recently urbanized areas have more native vegetation as compared to older urbanized areas, which have a higher density of buildings and more isolated habitat fragments (Bolger et al., 1997). The urban sites included in this study were all within areas that were developed within the past 20 years (Hurtado & Mabry, 2019), so these urban parks with native vegetation may be similar enough to wildlands in environmental attributes that Merriam's kangaroo rats can persist. The trapping periods used for population estimation were too short to allow us to estimate survival in urban versus wildland habitats.

We tested over 200 animals from both urban and wildland sites for parasite presence, and found that urbanization was not associated with parasite infection of individuals or population‐level parasite prevalence. Although we had a large sample size of individuals, those individuals comprised just five populations from each habitat type for analyses of parasite prevalence; it is possible that increased replication may have detected differences in prevalence. However, a recent meta‐analysis (Werner & Nunn, 2020) found no difference between urban and rural environments in parasite prevalence in rodent hosts, suggesting that the typical expectation that urbanization will lead to an increase in parasitism may not hold for this taxonomic group. Further, Werner and Nunn (2020) suggest that factors including simple versus complex parasite life cycles and other complexities of particular host/parasite interactions can have a large effect on whether urbanization leads to an increase in parasitism. Although little is known about the life cycles of the parasite species examined in this study, other species in both the genera *Pterygodermatites* and *Mastophorus* have insects as their intermediate hosts (Luong & Hudson, 2012). These insects are then presumably consumed by Merriam's kangaroo rats (Decker et al., 2001). Differences in insect diversity and abundance in urban versus non‐urban areas have been documented (Bolger et al., 2000; Faeth et al., 2005; McIntyre, 2000) and attributed to pollution, alteration to water resources, and an increase in insect predators (Faeth et al., 2005; McIntyre, 2000). However, we did not collect data on insect diversity or abundance; furthermore, the specific insect intermediate hosts are currently unknown for *P*. *dipodomis* and *M*. *dipodomis*. Thus, we are unable to examine the effects of either insect diversity or abundance on parasite infection of kangaroo rats at the individual or the population level.

Finally, in some systems, bolder and more aggressive animals have been shown to have increased infections and/or be involved in a higher number of transmissions (Dizney & Dearing, 2013; Natoli et al., 2005). However, in a previous study conducted with animals from the same sites, we found that there was no difference in either boldness or aggression between urban and wildland kangaroo rats (Hurtado & Mabry, 2017), suggesting that these behaviors were not likely to influence individual‐level infection rates. A relatively small sample size of animals that were both infected with parasites and included in behavior trials precluded us from examining the relationship between parasite infection and behavior.

The lack of detected effects of urbanization on behavior (Hurtado & Mabry, 2017), population density, and parasite infection (this study) in combination with the positive effects of urbanization on body condition of kangaroo rats infected with *P*. *dipodomis* (this study) should not be taken as an indication that the effects of urbanization on this native rodent species are all positive or equivocal. Our studies have taken place in urban parks that retain native vegetation; obviously, land that has been developed into impervious surface, for example, is unavailable as habitat for native rodents. While population densities were comparable between urban and wildland study sites, the urban sites consisted of patches of native vegetation embedded within urban development, while wildland sites were surrounded by continuous suitable habitat for kangaroo rats, and presumably, rodent densities were similar across these unsampled areas. Further, in a related study, we found evidence of genetic structure in the Merriam's kangaroo rat populations studied here: populations within urban parks had reduced genetic diversity as compared to wildland populations and were genetically differentiated from each other and from wildland populations, indicating that even relatively recent urban development can have negative impacts on wildlife (Hurtado & Mabry, 2019). Taken together, the results of this series of studies indicate that the effects of expanding urbanization on native small mammals in the southwestern USA may be complex, yet also subtle, and that further studies are warranted as development increases.

## CONFLICT OF INTEREST

The authors declare no conflict of interest.

## AUTHOR CONTRIBUTIONS

**Gizelle Hurtado:** Conceptualization (lead); Data curation (equal); Formal analysis (equal); Funding acquisition (lead); Investigation (lead); Project administration (lead); Visualization (lead); Writing‐original draft (lead); Writing‐review & editing (equal). **Ghislaine Mayer:** Investigation (supporting); Resources (equal); Writing‐review & editing (equal). **Karen E. Mabry:** Conceptualization (supporting); Data curation (equal); Formal analysis (equal); Supervision (lead); Visualization (supporting); Writing‐review & editing (equal).

## Data Availability

All data are available at Dryad: https://doi.org/10.5061/dryad.8pk0p2nns.
